# Short-term memory depends on the level of emotional burnout

**DOI:** 10.1192/j.eurpsy.2024.780

**Published:** 2024-08-27

**Authors:** S. Tukaiev, I. Zyma, M. Makarchuk

**Affiliations:** ^1^Institute of Biology and Medicine, Taras Shevchenko National University of Kyiv, Kyiv, Ukraine; ^2^Faculty of Communication, Culture, and Society, Institute of Public Health, Università della Svizzera italiana, Lugano, Switzerland

## Abstract

**Introduction:**

Emotional burnout refers to a syndrome caused by chronic stress. The formation of emotional burnout may lead to persistent changes in cognitive activity and particularly in memory and attention.

**Objectives:**

As the power of human EEG-spectrum components varies significantly under cognitive testing, the aim of our study was to investigate the dynamics of changes of EEG parameters under a memory task depending on the severity of burnout.

**Methods:**

42 healthy volunteers (students aged 18 to 24 years) participated in this study. EEG was registered over a period of 3 minutes during the rest state and 10 minutes during a verbal memory task. The spectral power density (SPD) of all frequencies from 0.2 to 35 Hz was estimated. The Mann-Witney criterion was carried out for the comparison of the independent data samples. The correlations were estimated using the Spearmen’s coefficient correlation. In order to determine the stages of burnout we used the test “Syndrome of emotional burnout” (by Boyko), adapted for students.

**Results:**

We observed variations in parameters of EEG during memorizing and retention phases depending on the intensity of the burnout. The intensity of the Exhaustion stage varied inversely with SPD in alpha3 (parietal and temporal regions), beta1 (parietal regions) and beta2 (parietal, right occipital and temporal regions) during the memorizing phase. The formation of the Exhaustion stage of burnout was accompanied by a decrease in alpha3 (parietal, left occipital and right temporal regions), beta1 (parietal, occipital and left temporal regions) and beta2 (parietal regions) during the retention phase.

**Conclusions:**

Our data indicate that short-term memory depends on the emotional state of subjects.

**Disclosure of Interest:**

None Declared

